# Perspective on Lessons Not Learned: From Coley’s Toxins to Microbial Drug Delivery, Guidance for Institutional Review Boards (IRBs)

**DOI:** 10.3390/ijms27135985

**Published:** 2026-07-03

**Authors:** Brian P. Hanley, Alejandro J. Betancourt, Gustavo Gross, Wilbur (Bo) Bowne

**Affiliations:** 1 UTRGV Department of Surgery, UTRGV School of Medicine, 1210 W. Schunior St., Edinburg, TX 78541, USA; 2 Neurosurgery, Valley Baptist Medical Center, 1040 W. Jefferson St., Brownsville, TX 78520, USA

**Keywords:** brain neoplasms, glioma, tumors, brain tumors, sepsis, septicemia, bacterial cancer treatment, institutional review board, Coley’s toxins

## Abstract

The bacterial and toxin methods of cancer treatment date back 130 years. This paradigm rests on nonspecific bacterial-toxin-generated immunotherapy. This high-risk oncology research is experiencing a renaissance of methods that are among the most effective yet. Glioblastomas and other resistant cancers are the modern touchpoint, because of remission history following sepsis. Spurred by recent research deaths, we discuss protocols IRBs should consider in live bacterial or synthetic immuno-stimulatory trials. Human systemic inflammatory response syndrome immunology is unique due to non-functioning SIGLEC-13 and 17, which control excessive Toll-like receptor 4 (TLR-4) signaling. This is not a technicality like human CD8+/CD4+ T cells. SIGLEC-13&17 consequences are profound; humans are ≈330–200,000 times more sensitive to LPS/endotoxin than mice and rats. This human TLR-4 difference also applies to gene therapy and should inform the results from any animal model, including non-human primates. Clinical TLR-4 stimulation takes two forms: bacterial infection and sterile TLR-4 stimulators, and treatments differ. The stereotactic injection of calculated amounts of adjuvants like endotoxin, venoms/components, or synthetic alternatives may be safer than live bacteria. Inadequate planning for risk elements, basic predictive models, and treatments will likely cause death.

## 1. Introduction

*“…bacterial-based therapies far outperform more traditional methods. [for resistant cancers]”*

Meng et al. [1]

There is a renaissance in bacterial=based therapies for resistant cancers [1]. Consequently, institutional review boards (IRBs) are faced with a conundrum—how to reasonably protect patient safety without blunting the effectiveness of protocols that make use of bacterial and toxin methods that can result in sepsis or, in some cases seek, to put subjects into a cytokine storm or septic state and have them recover. These protocols are high risk; thus, this perspective was precipitated by this conundrum. Here we also cite gene therapy trials that appear to be vector-dose-related [2,3,4,5,6].

We are also informed by an innovative treatment that used injections of live bacteria into gliomas in an attempt to induce remission and to repeat a method first successful on cancer in 1891 [7,8]. That attempt did not succeed. Subjects became septic and died. In the resulting investigation, a survey was done, and, out of some 15 clinicians and scientists questioned not connected with the investigators, neither clinicians working on microbial methods nor scientists performing animal model studies of microbial methods were aware of the SIGLEC-13&17 mechanism discussed here nor its significance. Our group also noted similarities to continuing problems in gene therapy trials in which subjects periodically die.

The principal investigators in the aforementioned innovative treatments had done proper rat experiments, and used that data to guide their trial with the extensive work of Coley [7,8], an inspiration. The investigators performed their human treatment study under local innovative treatment rules at the suggestion of the FDA (see Appendix A). This is relevant because other clinicians may find themselves in a similar situation. Perhaps aware of remission case reports or the work of Coley, other clinicians might take up a project from an animal study that will not be understood to not directly apply to humans and similarly fail. Without putting wisdom gained into the literature, the field cannot benefit from these tragic events.

The concept of this review began in the hope that such subject deaths could be learned from. We are aware that the underlying unique aspect of human biology that underlies these deaths remains rarely taught. We know of no immunology program that specifically teaches this SIGLEC-13&17 issue and human endotoxin sensitivity. We hope to spur remedying that problem and want to provide guidance for others interested in this area of oncology as well as the dose–response to gene therapy vectors. We ask our readers to receive this in the spirit of improving medicine by education.

## 2. Sepsis, Remission in Cancer, and Bacterial Methods

**Figure 1:**
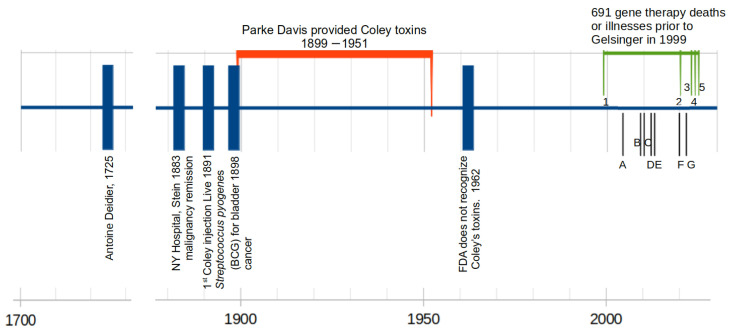
Timeline. Large blue bars: Coley (Deidier’s 1725 thesis noted inverse cancer relation [14], other time points from Nauts [7,8]) and McCarthy [13] Red timeline: Time span of Coley’s toxins as medication. Green timeline, upper right: Selected gene therapy cases (1—Gelsinger [15], 2—Audentes [4], 3—Astellas [5], 4—Pfizer Duchenne [6], 5—Sarepta [3]). Black timepoint bars upper right: Selected microbial therapies (A—*Clostridium Novy-NT* bacteriolytic [16], B—*Listeria*-based breast cancer vaccine [17], B—*Listeria* vaccine for cervical cancer [18], C—bacteria for targeted drug delivery [19], D—oncolytic virus review [20], E—*Salmonella X-CD20* for lymphomas [21], F—AAV DNAse-1 restores local immunity colorectal cancer model [22], G—bacteria in bioimaging & diagnostics [23]).

Because glioblastoma tumors are most resistant to treatment and have low survival rates, with few therapeutic options, these tumors provoke medicine to strike out in any direction that might be helpful. Thus, glioblastoma is a primary focus here. There have been persistent observations of an association between survival of sepsis and remission of glioblastoma [9,10,11] (Figure 1). These cited cases join the over-200-year history of neoplasms going into remission after severe infection, which was the original reason for William B. Coley trying bacteria in cancer treatment. Nauts et al. [7,8] reviewed the early 20th century work of Coley, who pioneered systematic use of killed microbial preparations to treat solid tumors, and is recognized as the father of cancer immunotherapy [12]. The first reference is a detailed discussion of most of Coley’s work and contains the contents of his formulations just above the References Section [7] (pp. 93–96). All but one of Coley’s formulations were killed bacteria: *Streptococcus pyogenes* and *S. pyogenes* combined with *Serratia marascens*, known as Coley’s toxins. The second Nauts reference provides a well-organized summary of effects, effectiveness, dose and route methods, frequency of dose and duration [8]. Coley’s very early conclusion was that deliberate inoculation with live bacteria was too dangerous. Hence Coley’s toxins became a pharmaceutical product of Parke-Davis from 1899 to 1951 [13]. Part of the problem with Coley’s toxins was the lack of standardization and assays to make them properly reproducible. This should no longer be a problem today.

Edvokimova et al. also reviewed Coley’s work, discussing it as a cancer vaccine [24], which coincides with our independently coined term ‘in-situ vaccination’ to describe a mechanism of activity.

The bacterial renaissance includes a host of methods, from unmodified live bacteria to genetically engineered for receptors, toxin production, and nanoparticles, which are discussed in a review by Meng et al. [1]. Meng also references issues with bacterial treatments, citing “*toxicity, DNA instability, unchecked growth, ineffective targeting,* [and] *off-target infection*” [25,26], but does not address these issues as we do here.

In addition to surgery and radiation, glioblastomas and other tumors are the object of a great deal of research into therapeutic agents ranging from small molecules to toxin-fused cytokines [27]. Since such patients are terminal, novel approaches with promise need to be vigorously pursued.

Indications of bacteria have been found in glial cells in non-pathogenic human brains, although whether they are beneficial, harmful or benign is unknown. However, this condition may be related to neurological disorders, and a route from the gut to the brain may be via the vagus nerve [28,29].

Bacteria and viruses injected intravenously as therapeutics for oncology can migrate to and replicate in tumors that appear to act as immune privileged sites [30]. The bacterial agent Clostridium novyi-NT (a strict anaerobe with toxin deleted) has shown positive results in an animal model [16]. This bacterial system only reproduces in the anoxic interior of tumors; consequently, it leaves a rim as well as small metastases untouched. Salmonella expressing CD20 antibodies and a drug conversion enzyme have shown effectiveness against lymphoma [21]. Oncolytic viruses are a well-established area of research [20].

Research into vaccination against cancer has mostly focused on identification of over-expressed self-antigens as vaccination targets [31]. Listeria as a vaccination vector has shown success in a rodent model [32], and attenuated Listeria has shown clinical safety in humans, but the anti-tumor response was poor [18].

Microbes can act directly, they can be vectors to deliver anti-tumor agents, and they can have indirect effects such as the Bacillus Calmette–Guérin (BCG) tuberculosis vaccine, which sensitizes cancerous cells to radiation [33]. There is a direct effect of a bacterium or virus attacking tumor tissue when injected; though, without genetic engineering of the microbes, bacterial destruction of tissue is nonspecific to the tumor. Such destruction and an immune-privileged environment inside the tumor that provides a haven for microbes probably explain some clinical observations of the anti-tumor efficacy of infection. However, observation of post-recovery remission after clearance of infection can only rarely be due exclusively to that direct effect, as full remission could only occur if the entire tumor was eradicated.

There are several mechanisms that can be involved in the infection-induced remission of a tumor. (A) Direct destruction of cancerous cells by infective bacteria. (B) Profound activation of immune system by cytokines drawing natural killer (NK) cells, neutrophils and macrophages to the site due to intra-tumor injection. (C) The systemic effect of pushing the immune system into an over-stimulated state. This mechanism is related to what checkpoint inhibitors are designed to do. (D) Antibodies developed against antigen in tumor. (E) Activation of cytotoxic T lymphocytes (CTLs) sensitized to tumor antigens, which is what CAR-T therapies do synthetically.

## 3. Immunology of Bacterial Preparations in the Context of Cancer Treatment

**Figure 2:**
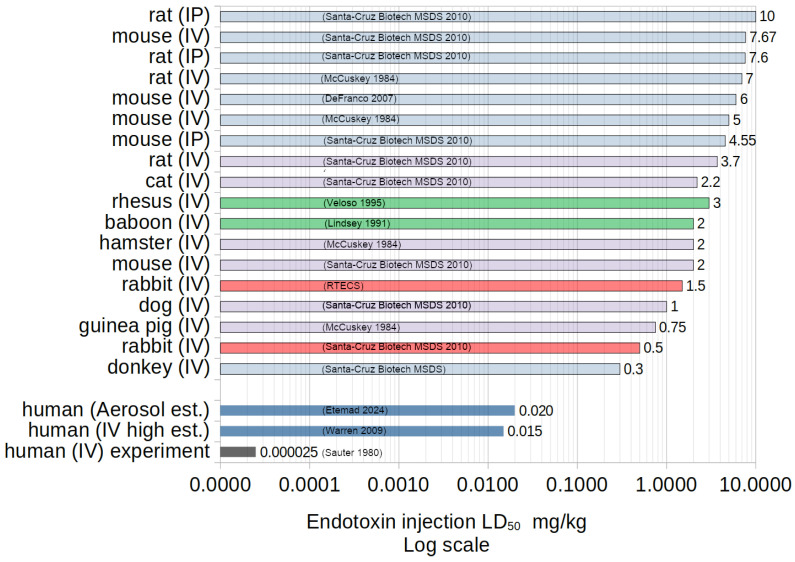
Log scale LD_50_ for standard endotoxin (LPS) doses. See Appendix B [36,37,38,39,40,41,42,43,44]. For humans, estimates are not well-characterized due to ethics issues. The lowest human dose (dark gray bar) of 25 ng/kg may be the most reliable dose. High human doses are estimated (blue bars). The closest animals to humans, baboon and rhesus (green bars), are hundreds to thousands of times less sensitive to endotoxin than humans (blue and dark gray bars). Rabbits (red bars) have been the standard endotoxin animal model, and they are tens to thousands of times less sensitive than humans.

Three differences between rodent animal models and human clinical cancer are significant. First, cancer models are in-lab strains of rodents, many of which have immune system defects—their cancers are not spontaneously occurring [34]. Since sepsis is an immune system overstimulation effect, using bacteria in animal models that have far greater tolerance for endotoxin may show greater efficacy due to the artificial nature of the tumors. Second, human treatment may encounter patients somewhat immune compromised by chemotherapy, radiation, or other factors. This can make them less capable of fighting infection; hence, when they do progress to sepsis, their clinical course can be more rapid and recovery more difficult [35]. Lastly and most important for microbial therapy is the extreme human susceptibility to sepsis relative to rodents, monkeys and even chimpanzees, shown in Figure 2, which presents data on estimated LPS dose range for humans relative to animals. There is no comparative data by strain in humans because such studies are unethical to conduct due to the intensity of human immune reactions. Estimates, when made, use nano-doses; animals are used for true LD_50_ estimation, with observations from clinical cases.

### 3.1. Cause of Sepsis

The cause of sepsis is commonly thought to be infection. However, technically speaking, sepsis is due to pathogen-associated molecular patterns (PAMPs) that the immune system is sensitive to. PAMPs trigger toll-like receptors (TLRs) [45], c-type lectin receptors (CLRs) [46], and cytoplasmic pattern recognition receptors (cPRRs) [47] (this last responds to nucleic acid motifs). In addition, damage-associated molecular patterns (DAMPs) from cellular debris also trigger inflammatory cytokines [48].

The root cause of sepsis can be PAMPs from bacteria, fungi or viruses, but it is usually triggered by broken-down bacterial cell walls, technically referred to as endotoxin or lipopolysaccharide (LPS) from Gram(−) bacteria [44]. LPS binds to toll-like receptor 4 (TLR-4) [49]. Thus, a standard laboratory model of sepsis is injection of sterile LPS. This can also duplicate multi-organ failure, which may be triggered by DAMP overstimulation also through TLR-4.

### 3.2. Difference in Sepsis Development in Animals and Humans

There is a radical difference in susceptibility to cytokine storm from TLR-4 stimulation in humans versus animals. Figure 2 shows relative doses [36,37,38,39,40,41,42,43,44].

Normal LPS LD_50_ in 25 g mice is approximately 150 µg (6 mg/kg), taking 35 h to complete its course. In mice, the LD_50_ of LPS depends on liver protein synthesis. However, in mice pretreated with β-galactosamine to inhibit liver protein synthesis, the mouse LD_50_ is approximately 5 ng (200 ng/kg), 30,000 times less [50]. While LPS sensitivity in mice may be phenotypically tunable to appear similar to human sensitivity, the mechanism is different and hence unreliable.

By comparison, normal humans show reactions at 2–4 ng per kg (Figure 2). The human LD_50_ for LPS is 25 ng to 15 µg per kg, two to five orders of magnitude lower than the mouse mean. Humans are 6000 times larger yet die at doses only 0.02 to 11 times the dose that kills a 25 g mouse. If humans had the immune systems of mice or rats, our LPS LD_50_ would be around 900 mg for an average adult instead of 25 ng–15 µg.

### 3.3. Mechanism of Human LPS Sensitivity: SIGLEC-13 and SIGLEC-17

SIGLECs have complex roles that, in the context of toll-like receptors (TLRs), are normally regulatory [51,52]. Human LPS sensitivity is due to inactivation mutations of primate sialic acid binding Ig-like lectins (SIGLECs) for SIGLEC-13 and SIGLEC-17 [53,54]. Phenotypically, knockouts of SIGLECs produce hyper-reactive immune system components. The missing SIGLEC-13&17 active in other primates affect control over TLR-4 [53]. TLR-4 is the receptor for LPS [48]. TLR-4 is also the target for HMGB1 in DAMP signaling, which occurs with tissue destruction [55]. Thus, once the cycle starts and amplifies to self-tissue destruction, it is self-reinforcing, requiring immediate intervention to stop the self-destructive cycle. TLRs also have crosstalk between them through various pathways [56,57,58,59,60].

### 3.4. Animal Models

Currently, there are no SIGLEC-13&17 knockout animals to model this rather critical aspect of the human immune system. Rabbits have an LD_50_ of ≈500 µg/kg (still tens to thousands of times the human estimates), which has made them a test animal for certain preparations that could contain LPS.

Rodent SIGLECS map differently than primates due to the roughly 75–90 million years to a common ancestor. We have worked on the animal model problem and found that mouse and rat immune systems are so divergent in this respect due to millions of years of evolutionary distance that homology does not exist in a way that could be relied on. So, our proposal is to use a non-human primate model, which is considerably more difficult than mouse or rat models. There are early immune organoids available [61,62]. However, an organoid does not necessarily demonstrate the breakdown from LPS that an intact animal does, and human monocytes respond similarly to mouse monocytes in vitro [37].

## 4. Protocol Elements in Live Bacterial Inoculation in Humans

When sepsis occurs naturally, the subject’s response may result in recovery; however, mortality is very high [63], and cancer patients have mortality 2.5–2.7 times above the usual mortality of sepsis with treatment [35].

If an investigator conducts a rat or mouse study of live bacterial inoculation, rats and mice will not usually require antibiotics for recovery. Thus, those conducting such studies may believe that withholding antibiotics will simply ensure sepsis induction rather than end human lives.

With this preamble we present protocol elements that would optimally be present. Such a protocol should take advantage of the most effective treatments for septicemia and have decision points for use.

### 4.1. Decision Points and Response Infrastructure

Decision point criteria and response(s) to be undertaken should be present. Attention to the information packet attending the subject, with 24 h contact availability, can help obviate problems arising from subjects being treated by emergency department (ED) physicians and nursing staff that are not enrolled in the study and hence are naïve to the protocol. Protocol-naïve medical treatment of clinical trial subjects in the ED happens for multiple reasons. Such problems can cause patient death, which has terminated promising medical products unnecessarily in the past. Problems with treatment of clinical trial crises by protocol-naïve ED or other medical staff are why many clinical trials bring subjects into their own treatment centers to ensure proper phase 2 trial execution. It should be kept in mind that strikes and other exigent circumstances may occur, leaving the research team as the fallback. All such events should be recorded carefully and reviewed, because phase 3 trials will not usually be conducted in the researcher’s specialized facilities. It is not just the narrowly defined science of a specific product that determines viability of a product in a clinical trial; it is the entire clinical trial design and operation.

If it is not considered practical for the trial to house and treat subjects entirely within their facilities, then special attention to contact availability of clinical trial staff, pre-contact discussion with the EDs likely to receive a subject, and practical steps such as wrist bracelets that EMT services can access with contact information should be considered. Trials could consider making use of wearable monitors that continuously report as a safety feature.

An ancillary factor is that practicing physicians, and particularly surgeons, have an orientation to immediate action for the sake of patients. This can be a source of significant friction between research scientists, surgeons, and clinical trial medical staff. There are valid reasons for these differences. It is helpful to clarify the decision-making process beforehand relative to the clinical care of subjects when there are multiple interested parties. In a hospital, teams have the attending physician. A similar system is advisable to ensure the identification of the responsible party.

### 4.2. Sizing of Likely Time Window

When septicemia, or flirting with septicemia, is the (temporary) goal of a live bacteria protocol, rapid progression and high risk of mortality should be expected in absence of aggressive treatment. In the plan, there should be a timeline for the replication rate of the bacteria selected and typically an exponential calculation model of the expected time window based on inoculation. High-risk protocols such as this should take advantage of the ability to perform the procedure more than once, with an initial dosing and treatment schedule that should be quite safe. One can always raise the dose or push the response envelope further next round.

### 4.3. Diagnostic Monitoring

There should be in place a comprehensive set of tests that will be conducted and monitored alongside study parameters for patient safety. We suggest the current NIH-published CME book on systemic inflammatory response syndrome (SIRS) [64], in its entirety, as the starting point.

SIRS is defined by the presence of any two of the following criteria:

- Body temperature > 100.4 °F (38 °C) or <96.8 °F (36 °C);
- Heart rate > 90 bpm;
- Respiratory rate > 20 breaths/min or PaCO_2_ < 32 mm Hg (4.3 kPa);
- Leukocyte count > 12,000/μL, <4000/μL, or >10% immature forms (bands).

Specific trials may have additional criteria and diagnostics. A trial’s decision points are likely to vary from this basic list, with higher body temperatures in particular desired.

Attention should be given to the rate of change and the expected curves when things have gone too far. Consideration should be made for subject populations, as it may be necessary to respond differently based on other chemotherapy, medical history and comorbidities.

There should be consideration of repletion of gut microbiome for subjects treated with antibiotics, and this factor may be significant for long-term outcome. It will not be possible to eliminate this as a potential confounder, but attention and diagnostic testing following this element are appropriate.

### 4.4. Antibiotics

Human patients inoculated with robust bacteria will require antibiotics. Thus, careful selection of a set of antibiotics effective against the bacterial strain chosen is the most obvious first layer of safety. In the plan, there should be decision point(s) when antibiotics should be applied, dose, any recommended combinations, and route (i.e., oral, subcutaneous injection, intravenous, intrathecal, combination, or other method(s)).

Depending on the bacterial strain, specific antibiotics should be available, with attention to the antibiotic mechanism. For example, Polymyxin B sulfate can attenuate sepsis by directly combining with lipopolysaccharide (LPS, also known as endotoxin) [65]. However, the LPSs from different bacterial species varies a great deal, and this antibiotic does not bind equally well to all varieties of LPS, so in a deliberate inoculation, the efficacy of Polymyxin B sulfate (and other antibiotics) by dose on the chosen bacterium’s LPS would be a helpful test result. Where an antibiotic may be recommended under special circumstances and doses, such as Polymyxin B sulfate, that may exceed guidelines, a brief explanation of the rationale can give a protocol-naïve physician more confidence in applying a protocol’s recommendations.

### 4.5. Immune Modulators

Drugs that act on the immune circuit range from TNF-α inhibitors to IL-10, small molecules and steroids. In live bacterial inoculation/infection, use of most such treatments lowers survival. However, in the short term, to treat a septic crisis, temporary use may be warranted. Medicine uses corticosteroids short term in septic crisis now and has experience with immune-compromised patients, and steroids would generally be a first choice.

### 4.6. Antitoxins

For a bacterial treatment or a synthetic simulation, toxins other than, or in addition to, endotoxin may be present by design. As an added layer of safety, antibodies to certain toxins could be developed for use on subjects. Fragment antigen-binding (Fab) anti-toxins similar to antivenins can be developed [66], as well as more advanced techniques [67,68]. We note that such preparations also need to test for endotoxin, as serum extractions have significant issues because animal serum is an excellent growth medium, and one-off lab-produced material may not have the quality control procedures of the pharmaceutical industry. We provide this reminder to investigators because, again, what may test just fine in animals may be quite problematic in humans.

### 4.7. Ghrelin

Ghrelin has shown efficacy at treating sepsis [69,70,71,72], stabilizing animals quite effectively. With the caveat that humans are significantly more sensitive, this could be a treatment modality in an experimental procedure. There have been many human clinical trials registered for ghrelin, though none for treatment of sepsis. Thus, ghrelin is an experimental treatment that should be available within an IRB/IND application, and the literature available indicates ghrelin is safe and should be effective for the treatment of sepsis. Current law requires the FDA not to block access to potentially life-saving drugs in terminal patients (though manufacturers are not required to provide them). Ghrelin, being unpatentable, can be ordered from compounding pharmacies.

### 4.8. Hyperbaric Oxygen

Hyperbaric oxygen (HBO_2_), with or without antibiotics, can be a life-saving treatment [73,74]. HBO_2_ activates the patient’s immune system to its maximum capability to fight infection. The metabolic activity in the cells throughout the body increase; the immune privilege granted to tumors by their anoxic interior ends during oxygen saturation. Similarly, the high pressure easily overcomes the increased interstitial pressure of tumor cells that keeps chemotherapy molecules out. Increased metabolism raises motility of leukocytes and greatly improves oxidative killing of bacteria by neutrophils. The liver can raise the rate of destruction of endotoxin. HBO_2_ is underutilized for treatment of infection and utilized late when it is.

The improved activation and motility of CTLs with HBO_2_ may also positively impact the results of a study relative to cancer remission.

HBO_2_ should be part of a clinical trial featuring bacteria with high risk of infection. For a subject in crisis, after antibiotics and any other medications are applied, HBO_2_ should be provided as soon as possible.

### 4.9. Surgical Intervention

Together with other treatment modalities, surgical drainage/removal of endotoxin-containing/generating material, ‘source control’ (e.g., pus or infected tissue), can relieve the body of the LPS burden (and other bacterial toxins) that over-stimulates the immune system.

## 5. Synthetic Induction of Septic-like Cytokine Storm

Coley’s work, the pioneering reference, concluded that killed bacteria were safe enough to use and discommended live cultures. However, this fact and the formulas are buried at the end of the primary Nauts et al. paper, which is 103 pages long. Modern paper format is biased to a few thousand words, where pre-internet standards for major reviews could be short-book length. Essentially, Coley’s 13 toxin formulas [7] (II–XIV, pp. 93–96), [8] (p. 484) are super-adjuvants with similarity to Freund’s complete adjuvant (FCA) [75,76]. FCA is an emulsion of oil, water and inactivated mycobacteria that causes intense immune activation and can be used in animals to model autoimmune disorders.

We do not mean to suggest by our discussion that only Coley’s formulas should be used or emulated. We use Coley’s formulas as a jumping off point for discussion because they are so prominent in the literature.

### 5.1. Characterization of Injectable

Coley’s toxins suffered from difficulties with assays and creating a product that would have reliable replication of effect. Today, it is possible to characterize the mixture with a high degree of accuracy. The limulus amebocyte lysate (LAL) test is the standard for endotoxin, with the caveat that the LAL test has different responses to different bacteria.

Where bacteria are involved, characterization and confirmation of the species should be done, typically by nucleic acid methods. Specific toxins should be known, and if possible an assay should be used to characterize and quantify.

### 5.2. In Situ Vaccination and Stereotactic Placement of Injection

Coley’s formulas II-XIV contain inactivated bacteria that would cause intense immune activation; thus, it is logical to believe that a significant mechanism of his formulas worked by activating the immune system in the vicinity of the tumor, and this sensitizes the immune system to cancerous cell antigens. We coined the term in situ vaccination as a mechanism by which tumors might go into remission. In situ vaccination should be performed with controlled dosage of specifically selected materials using stereotactic injection or careful manual placement.

### 5.3. Venom-Based Materials

Venom-based materials can accomplish debulking by enzymatic action [77,78]. This enzymatic digestion of tissue also presents self-tissue to the activated immune system and should result in HMGB1 DAMP signaling via TLR-4. Note that snake venoms have very high self-adjuvancy, possibly greater than FCA [79].

### 5.4. Gene Therapy and CRISPR Toxicity

The CRISPR cell toxicity issue wherein p53 is triggered to cause apoptosis [80,81] is a DAMP signaling mechanism and can be treated as a synthetic cytokine storm induction. It is worth noting that in vivo and invitro, CRISPR without suppression of p53 selects for p53 mutations that are cancer precursors [82]. Thus, p53 suppressors [83] should make both the CRISPR toxicity issue and the selection problem avoidable.

## 6. Post-Treatment of Synthetic Induction

Synthetic cytokine storm induction includes dose side effects of gene therapy vectors [2,3,4,5,6], and synthetic induction has treatment options not as available to live bacterial inoculation, thus providing a high margin of safety.

TNF-α inhibitors, such as adalimumab, etanercept, infliximab [84], and small molecules such as DOI [85] can rescue by interrupting the “feedback scream” that is synthetic sepsis. Similarly, dimethyloxallylglycine (DMOG), a small molecule, may be helpful [86]. Injection with IL-10 is protective against injection of LPS, and survival in the presence of an infection is also improved by antagonists to IL-10 [87]. Corticosteroids are an option to respond to synthetic induction that physicians are familiar with; however, they may not be effective enough in some cases. There are excellent medications available today for reining in synthetic immune system over-activation. In many cases, one injection of a TNF- α inhibitor should be sufficient to terminate the syndrome, as most TLR-4 stimulants process out of the body in 36 h.

## 7. Discussion

Live microbial oncotherapies have serious concerns. Before human clinical application of a live bacterial or viral treatment, a model of how the immune system is expected to respond should be defined. This should include attention to immune-privileged sites. Immunology is complex enough that there are bound to be surprises, but today’s immunology is quite sophisticated. Animal studies should take into account that human susceptibility to sepsis is hundreds to hundreds of thousands of times greater than that of mouse models, and those who review these protocols should understand why. Since the primate SIGLEC-13&17 issue for humans is rarely taught in immunology curricula at this time, this issue remains a source of patient deaths in gene therapies and microbial approaches to cancer treatment.

A multi-tiered protocol for post-inoculation treatment with bacteria includes monitoring diagnostics, wide spectrum and specific antibiotics, Polymyxin B sulfate, hyperbaric oxygen, ghrelin and surgical drainage. The use of hyperbaric oxygen should be considered as a primary element of the protocol to maximize leukocyte activity. Potential use of neutralizing factors (for instance specific antibody) to bacterial toxins or a virus can be considered with care to validating that the antibody preparation does not contain significant amounts of endotoxin. Decision points should be defined for when each element of the protocol should be used.

Synthetic methods provide similar, if not identical, immune system stimulus as live bacteria, with near-perfect patient safety.

By targeting sets of toll-like receptors and the use of adjuvants [88,89], along with the application of toxins, safe simulation of infection can be provided to patients. Vaccine theory supports repetitive inoculation at intervals of 3 weeks [90], providing theory support to Nauts’s discussion of this in Section 2, Techniques of Administration [8] (p. 486). In addition, synthetic induction reactions can be utilized together with treatments such as HBO_2_ and oral immune stimulators [91] for synergistic effects.

## Data Availability

This research generated no new data not published herein.

## Protection of Identity

While not technically covered as Morbidity and Mortality discussion, we treat the specifics and identities related to the teaching instance discussed here in the same spirit, mentioning only what is strictly relevant to the points being made.

## Abbreviations

The following abbreviations are used in this manuscript:

BCG	Bacillus Calmette–Guérin (tuberculosis vaccine)
CLR	C-type lectin receptor
CTL	Cytotoxic T lymphocyte
CMO	Chief medical officer
DAMP	Damage-associated molecular pattern
DMOG	Dimethyloxallylglycine
DOD	Department of Defense
FCA	Freund’s complete adjuvant
FDA	Food and Drug Administration
FOIA	Freedom of information act
HBO_2_	Hyperbaric oxygen
HHS	Health and human services
HIPAA	Health Insurance Portability and Accountability Act of 1996
IND	Investigational new drug
IRB	Institutional review board
LD_50_	Lethal dose for 50%
LPS	Lipopolysaccharide
MOU	Memorandum of understanding
NK	Natural killer cells
PAMP	Pathogen-associated molecular pattern
SIGLEC	Sialic-acid-binding immunoglobulin-type lectins
TLR	Toll-like receptor
TNF	Tumor necrosis factor

## Appendix A. Innovative Treatment Rules

Innovative treatment rules allow physicians to perform medical procedures that they, and the institutions in which they work, see fit, provided that the medical procedures do not make use of critical study materials that are not FDA-approved that are sourced from out of state. This is due to the FDA’s jurisdiction being based on regulation of interstate commerce and the assumption that hospitals will make choices for potential patient benefit. For work using critical study materials produced within the state, conformance to FDA regulations is voluntary unless a state’s law specifies otherwise or a memorandum of understanding (MOU) exists. No such state laws or memoranda were known at the time of writing. States vary as to their requirements for completion of an institutional review board (IRB) process in such cases.

However, in recent years some case law on FDA jurisdiction has been upheld where previously innovative treatment rules were believed to apply. It should also be carefully noted that FDA IND approval provides legal protection for clinical trial investigators and their institutions against criminal and most civil actions, provided the rules are followed. Innovative treatment protocols do not have such protections. Providing innovative treatments outside of the FDA’s IND system has resulted in the investigators facing criminal charges, including homicide. In the instance referred to here, referral for charges was discussed.

The US department of defense (DoD) has voluntarily agreed to conform to FDA regulations via an MOU. The current Department of Defense MOU, with the FDA last revised in 1987, dates to 1974 [92]. A number of prominent academic institutions have MOUs with the FDA, although those do not strengthen the requirements for medical research [93].

## Appendix B. Lipopolysaccharide Lethal Dose Characterization

Determining human LPS LD_50_ is difficult for a host of reasons. Among the authorship, two of us published an extensive toxicity meta-analysis of 1003 lethal dose studies of snake venom, so we are quite familiar with issues of biological toxins [94]. That study showed that the primary correlate of LD_50_ range of toxicity was how many studies were conducted and that the LD_50_ range could span over 3 orders of magnitude. LPS studies are far sparser than venom studies but LPS shares some aspects with venom.

The structure of LPS varies between Gram(−) bacterial species, the amount of LPS per bacterium is maximized in the log phase of growth, and human TLR-4 receptors inevitably have mutations that could affect binding. Add to this that the physical condition of the human subject/patient can vary a great deal and has a strong effect on survival. On top of this, there is a PNAS study that shows subjects trained in the Wim Hoff method could control their innate immune response to LPS injection [95]. However, no further studies were conducted to attempt to determine limits of such control. We do know that lymph nodes are innervated and LPS receptors signal to those nerves, which is a likely mechanism.

In addition, here we are talking about acute dose survival, when recent work shows that 4-year mortality of sepsis survivors may be 75% [96].

The primary point to make is that for biological toxins that act on receptors, toxicity is complex, and the numbers should be expected to form a curve that for LPS requires many more studies than are likely to be performed. So, the numbers we present here are not firm, particularly for humans. What can be said is that humans are generally very fragile in this respect. We do not intend to suggest that response to LPS should be as wide as seen with snake venoms: we intend to convey that there is not enough data to say what the range really is.

Human LD_50_ studies are rare for any chemical due to ethics problems; thus, estimates are soft, and safety pushes to the low end for human estimates, where such estimates are made. Animal studies are harder to do today. In the past there was much less or even no oversight of animal studies. In today’s ethics climate, animal ethics approval can be as difficult to get as human study approval, and animal ethics committees may push back strongly on protocols that will kill the animals and cause them pain and suffering. We had to scrap a study of this kind 5 years ago that was refused on that basis.

LPS is no exception to stronger animal ethics, and unlike snake venoms, there has been no reason to fund departments of scientists across the globe to study LPS lethal dose characteristics in different strains of Gram(−) bacteria for the past century. Thus, the human experimental study by Sauter is a self-experiment in which the injected party was admitted to ICU for 8 h after injecting [36]. If this experiment was performed today, it would face obstacles to publication, although law and regulation almost entirely allow and even approve of self-experiments [97].

Sauter injected 2 µg of *E. coli* O 127:B8 LPS obtained from Sigma-Aldrich, St. Louis, MO, USA. Sauter did not report nanograms per kilogram, so we assumed that the party massed 80 kg. Thus, we present a value of 25 ng/kg in Figure 2.

The non-human data references in Figure 2 were collated starting with a Santa Cruz Biotech material safety data sheet produced by CHEMWATCH [39]. Other sources were acquired to expand those figures and to cross-validate that the range appeared to be reasonable [36,37,38,39,40,41,42,43,44]. In the future, it may be possible to monitor and record LPS levels in human blood and using those patient records produce a study with *N* in the thousands of cases to improve our understanding. For now, this is not available.
